# Fatal *Clostridium sordellii*-mediated hemorrhagic and necrotizing gastroenteropathy in a dog: case report

**DOI:** 10.1186/s12917-020-02362-y

**Published:** 2020-05-24

**Authors:** Paul Capewell, Angie Rupp, Manuel Fuentes, Michael McDonald, William Weir

**Affiliations:** College of Medical, Veterinary and Life Sciences, Institute of Biodiversity Animal Health and Comparative Medicine, University of Glasgow, Urquhart Building, 464 Bearsden Road, Glasgow, G61 1QH UK

**Keywords:** Bacterial toxins, *Clostridium sordellii*, *Clostridium perfringens*, Dog diseases, Genomics, Hemorrhagic gastroenteropathy, Hemorrhagic canine gastroenteritis

## Abstract

**Background:**

Canine hemorrhagic gastroenteritis (also *canine gastrointestinal hemorrhagic syndrome*) is commonly associated with *Clostridium perfringens*, although in some cases the etiology remains unclear. This report describes a fatal acute hemorrhagic and necrotizing gastroenteropathy in a dog associated with *Clostridium sordellii*, a bacterial species never before identified as the etiological agent of hemorrhagic and necrotizing gastroenteropathy in dogs.

**Case presentation:**

A fully vaccinated, eight-year-old, female neutered Labrador presented with a history of vomiting without diarrhea. Clinical examination revealed pink mucous membranes, adequate hydration, normothermia, and normocardia. The dog was discovered deceased the following day. Post-mortem examination showed moderate amounts of dark red, non-clotted fluid within the stomach that extended into the jejunum. Discoloration was noted in the gastric mucosa, liver, lungs, and kidneys, with small petechial hemorrhages present in the endocardium over the right heart base and thymic remnants. Histological analysis demonstrated that the gastric fundic mucosa, the pyloric region, small intestine, and large intestine exhibited superficial coagulative necrosis and were lined with a layer of short Gram-positive rods. Anaerobic culture of the gastric content revealed *C. sordellii* as the dominant bacterial species and neither *Salmonella* spp., *Campylobacter* spp., *C. perfringens*, nor *C. difficile* were isolated. Unexpectedly, whole genome sequencing of the *C. sordellii* isolate showed that it lacked the main plasmid-encoded virulence factors typical of the species, indicating that the genetic determinants of pathogenicity of this strain must be chromosomally encoded. Further phylogenetic analysis revealed it to be genetically similar to *C. sordellii* isolates associated with gastroenteric disease in livestock, indicating that the infection may have been acquired from the environment.

**Conclusions:**

This case demonstrates that *C. sordellii* can associate with a canine hemorrhagic and necrotizing gastroenteropathy in the absence of *C. perfringens* and illustrates the benefits of using bacterial whole genome sequencing to support pathological investigations in veterinary diagnostics. These data also update the molecular phylogeny of *C. sordellii*, indicating a possible pathogenic clade in the environment that is distinct from currently identified clades.

## Background

Canine hemorrhagic gastroenteritis (also *canine gastrointestinal hemorrhagic syndrome* or *acute hemorrhagic diarrhea syndrome*) is clinically characterized by (per) acute hemorrhagic diarrhea, frequently accompanied by vomiting and hemoconcentration. The disease is often associated with the presence of *Clostridium perfringens*, an aerobic, spore-forming, rod-shaped Gram-positive bacterium [1]. *C. perfringens* can produce several major toxins (Cpa, Cpb, Etx, and Iap/Iab) that are used to type infection (types A–E). However, the description of several new toxin genes (*netB*, *cpe*, *netE*, and *netF*) has recently expanded the range of toxinotypes [2]. In particular, isolates expressing both Cpe and NetF (termed type F) have been linked to acute cases of hemorrhagic diarrhea in canines and equines [1, 3–6]. This includes studies that identified the toxinotype in half of dogs presenting with acute hemorrhagic diarrhea [1, 6]. However, these reports contain several animals with hemorrhagic gastroenteritis and an uncharacterized clostridial infection, suggesting further pathogenic strains or species may contribute to disease*.*

*Clostridium sordellii* is an emerging pathogen of humans and animals that is commonly found in soil and sewage [7]. While many strains are non-pathogenic, some are virulent, particularly those expressing lethal toxin (TscL) and hemorrhagic toxic (TcsH) encoded on separate plasmids, pCS1 and pCS2 [7]. Humans are commonly infected at sites of soft tissue trauma and typically exhibit gas gangrene, edema, hypotension, absence of fever, tachycardia, intense leucocytosis, and hemoconcentration. Mortality is usually due to hypotension and multiorgan failure, likely mediated by capillary leak syndrome, septic processes, and toxic shock [8]. In contrast to this, cattle and sheep are more commonly infected orally and exhibit gastrointestinal disease and sudden death [9–12], whereas equines exhibit fatal internal omphalitis and atypical myopathy [13, 14]. Infection with *C. sordellii* has also been linked to incidences of necrotic enteritis in chickens [9].

TscL has been shown to be leucocidal, leading to lesions characterized by a profound absence or mild inflammatory responses [15]. TcsH is less commonly found and is hypothesized to cause alteration to the cytoskeleton, resulting in capillary leakage [10]. However, it is important to note that the majority of *C. sordellii* strains do not possess TscL nor TscH but are still pathogenic, albeit less so than those possessing these toxins [7, 16]. These strains do not contain any recognizable plasmids and additional chromosomally-encoded virulence factors have been suggested, including sordellilysin (Sdl), neuraminidase (NanS), and phospholipase C (Csp), for which the exact mechanisms of pathophysiology remain to be established [16].

## Case presentation

**Fig. 1:**
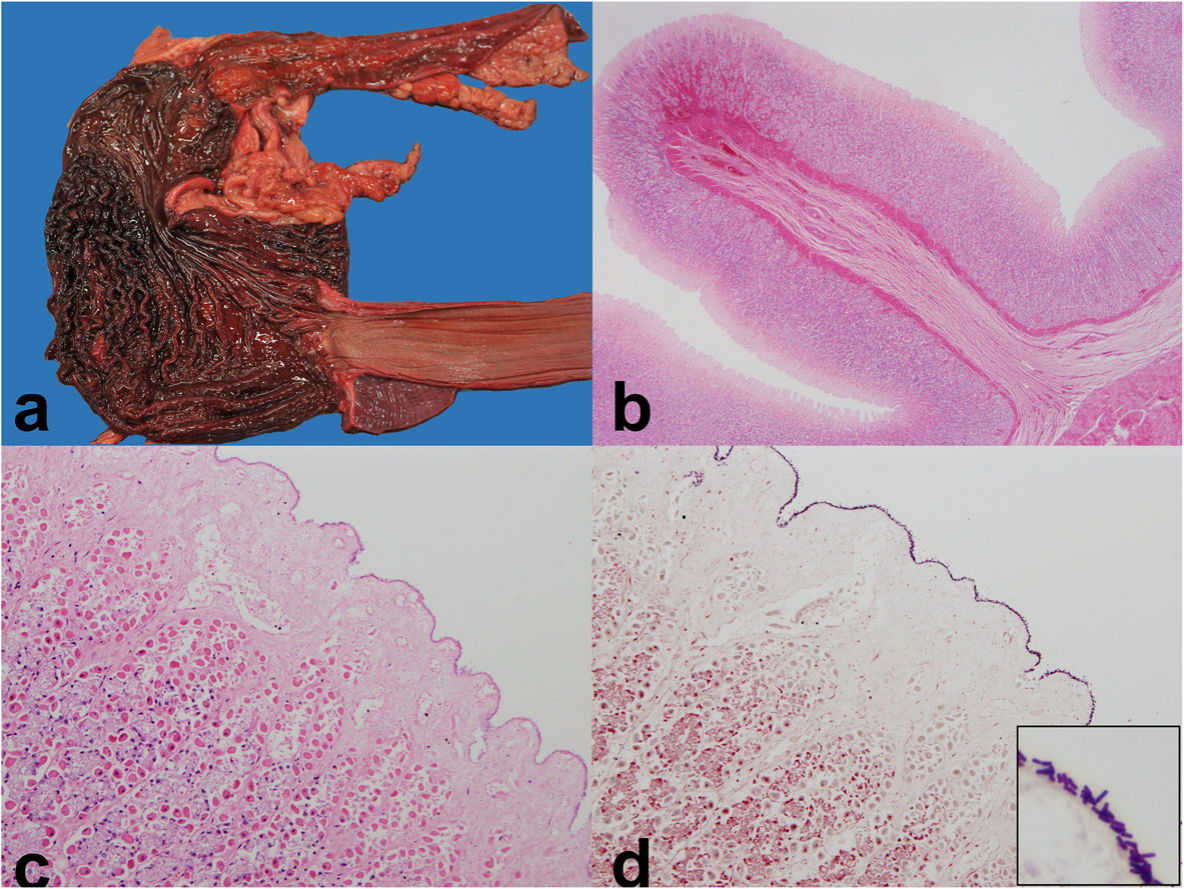
Gross and histological appearance of a canine stomach following infection with *C. sordellii*. The gastric mucosa was diffusely discolored dark red and the fundic region also exhibited irregular patches of more intense reddening (interpreted as hemorrhages) **a**. Hematoxylin and eosin stained tissue showed that the most proximal aspects of the fundic mucosa were necrotic and lacked appreciable inflammatory infiltrates (**b**-**c**). Small mucosal hemorrhages were present, and subsequent Gram-stain revealed that the mucosa was covered by a thin layer of small, Gram-positive rods (inset shows an enlarged view) (**d**)

In the present case, a fully vaccinated, eight-year-old, female neutered Labrador presented with a 24-h history of vomiting without diarrhea. Until this episode, the dog had been completely healthy, with a single vomiting episode reported approximately 3 months prior. It was noted that the dog was a known scavenger. Upon clinical examination, the dog presented with pink mucous membranes, adequate hydration, normothermia, and normocardia. The abdomen lacked any signs of bloating or dilation. The following morning the dog was found deceased. Gross examination conducted 7 h post-mortem revealed moderate amounts of dark red, non-clotted fluid within the stomach. The fluid extended caudally into the first two-thirds of the jejunum, whereas the remaining small intestine and large intestine were devoid of content. The gastric mucosa was diffusely discolored dark red, with the fundic mucosa containing additional irregular patches of more intense reddening that were interpreted as hemorrhages (Fig. 1a). The mucosa of the remaining gastrointestinal system was mildly reddened and this was interpreted as evidence of congestion. Liver, lungs, and kidneys were dark red, with small petechial hemorrhages present in the endocardium overlying the right heart base and thymic remnants.

Upon histological examination, the superficial third of the gastric fundic mucosa exhibited coagulative necrosis (Fig. 1b and c), and in a multifocal to coalescing distribution, the mucosal surface was lined by a thin layer of short Gram-positive rods (length: 6 μm; thickness: 1 μm; Fig. 1d), with small groups of rods also extending multifocally along the gastric pits into the necrotic layers. Inflammatory cells were not evident in any of the mural layers, although small multifocal hemorrhages were present in the mucosa. The proximal mucosa of the pyloric region, small intestine, and large intestine exhibited similar changes to those observed in the fundic region with these lesions mildly tapering towards a more multifocal to coalescing distribution in the large intestines and regions of more intense bacterial infiltrates and pronounced mucosal necrosis commonly associated with one another. Additionally, multifocal intra-alveolar and intra-bronchiolar hemorrhages were evident in the lungs and the adrenal cortex exhibited acute, multifocal to coalescing hemorrhages. Anaerobic culture of gastric content using horse blood agar revealed a profuse growth of a clostridial species that was subsequently identified as *C. sordellii* using the Analytic Profile Index (API) method (bioMérieux) and designated as strain 24,178. Aerobic culture using sheep blood agar revealed a profuse growth of *Cellulomonas/Microbacterium*, also identified by API. *Salmonella* spp., *Campylobacter* spp., *C. perfringens*, and *C. difficile* were not isolated.

**Fig. 2:**
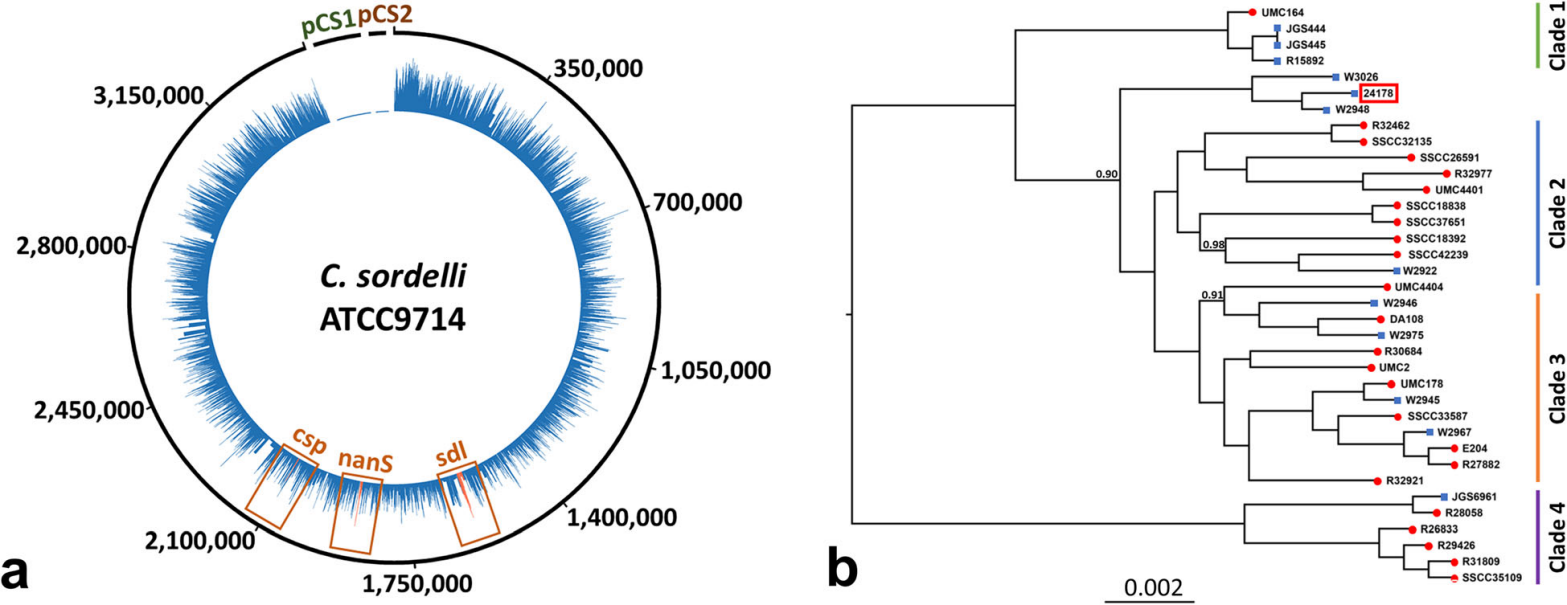
Genomic analysis of *C. sordellii* isolate. A schematic showing the depth of sequencing reads from strain 24,178 (blue) that mapped to the genome of *C. sordellii* reference strain ATCC9714 and the two toxin-encoding plasmids (pCS1 and pCS2). The numbers indicate base pair position. No reads aligned to pCS1 or pCS2 (highlighted in green and brown), although reads were found that mapped to the putative virulence factors highlighted in red; sordellilysin (*sdl*), neuraminidase (*nanS*), and phospholipase C (*csp*) (**a**). A cladogram of publicly available genomes for human (red circles) and veterinary isolates (blue squares) indicates that strain 24,178 (red box) is closely related to two livestock samples (W3026 and W2948). There is strong node support to suggest that this cluster does not sit within any of the four classical clades (support values are shown at the corresponding node if above 0.8 and the scale bar denotes nucleotide changes per position) (**b**)

A whole genome sequencing approach was utilized to fully characterize *C. sordellii* strain 24,178. To this end, purified DNA was prepared from culture using a QIAamp DNA Mini Kit (Qiagen GmbH, Hilden, Germany) and an aliquot containing 24 ng/ul was commercially sequenced using the Illumina HiSeq platform (MicrobesNG, Birmingham). This resulted in 378,245 read pairs aligned to a reference *C. sordellii* strain ATCC9714 that possesses both pCS1 and pCS2 plasmids. However, despite an average read depth of 18.1 across the genome, no reads were found to align to either plasmid, indicating that the *tscL* and *tscH* toxin genes were not present in the isolate under investigation (Fig. 2a). Sequence reads were assembled de novo using VelvetOptimiser for Velvet [17] and BLASTN in order to identify sequences similar to *tscL* and *tscH* in the resulting assembly and this also resulted in no hits, again confirming that the major toxin genes were not present. Further in silico plasmid detection using HyAsP [18] and plasmidSPAdes [19] detected no evidence of novel plasmids, whilst reciprocal BLASTN searches of the 123 de novo contigs assembled by Velvet against ATCC9714 also did not detect any regions that were unique to the isolate. The virulence associated genes sordellilysin (*sdl*), neuraminidase (*nanS*), and phospholipase C (*csp*) were all found to be present (Fig. 2b). The predicted sordellilysin amino acid sequence was identical to that found in other strains, while neuraminidase possessed a Leu397Ile mutation and phospholipase C featured an Asp480Glu mutation. These mutations have not previously been described and it is unknown what impact, if any, these would have on virulence. Finally, a core *C. sordellii* genome was created using 35 publicly available sequences and a previously established method [7]. This resulted in 1157 shared genes that were aligned using Mauve [20] to construct a phylogenetic tree with FastTree2 [21]. Similar to previous work [7], the tree was rooted with *C. difficile* strain R20291. The tree topology suggested that the isolated *C. sordellii* strain is closely related to two livestock *C. sordellii* strains (W3026 and W2948), possibly indicating that the dog contracted the infection via environmental ingestion. Neither of the closely related veterinary strains possess the plasmid-encoded toxins. While the four clades of *C. sordellii* were largely reconstructed in this analysis, the additional data provided by our analysis show that the new strain 24,178 and the related veterinary strains are placed within a distinct group between clade 1 and clades 2 and 3. This may suggest these strains are a novel environmental clade more closely related to the virulent clade 1 strains despite not possessing TcsL nor TcsH.

## Discussion and conclusions

This case report demonstrates that *C. sordellii* can cause a fatal hemorrhagic and necrotizing gastroenteropathy in dogs and should be considered as a potential causative agent for such syndromes alongside *C. perfringens*. The close relatedness of this isolate to strains previously recovered from diseased livestock suggests that the infection of this dog may have occurred via ingestion from the environment. *C. sordelli* infections associating with severe necrotizing gastroenteric disease have previously been described for several livestock species, including chickens [11], sheep [9], and cattle [12]. Fatal infections have also been reported for equines [13, 14]. Therefore, a history of scavenging or contact with livestock may represent a risk factor in such cases. It is notable that the strain of *C. sordellii* under investigation caused severe disease despite lacking the classical *tcsL* and *tcsH* virulence factors sometimes found in this species. Interestingly, the genetic basis for virulence associated with companion animal hemorrhagic gastroenteritis caused by *C. perfringens* has been the subject of recent investigations and this has resulted in the identification of the NetF toxin in isolates from canine [1, 4–6] and equine cases [4, 5]. This pore-forming cytotoxin co-locates with the *cpe* enterotoxin gene on a plasmid [4]. Since its discovery, large-scale studies have indicated a significant association with dogs with acute hemorrhagic diarrhea syndrome [1, 3–6] and a causal link has been proposed [1]. It is striking that we did not find any plasmid-encoded virulence factors in our strain of *C. sordellii*. While it is possible that the toxin-encoding plasmids were lost during culture adaption of isolate 24,178, as has been described for other isolates [7], the short-term growth in vitro and the close relationship between isolates 24,178, W3026, and W2948 suggest that this is unlikely. Our findings therefore highlight the need for further genomic studies to quantify the importance of chromosomally encoded virulence factors in this pathogen. Genetic characterization beyond the straightforward presence or absence of plasmid-encoded virulence factors may allow the pathogenic potential of an isolate to be estimated and an array of genetic polymorphisms associated with subtle pathogenic differences to be identified. An important aspect of this retrospective case study was the application of whole-genome sequencing to fully characterize the pathogen involved. As sequencing technology becomes a more readily accessible tool to support diagnostic veterinary clinical pathology, the use of predictive models designed to gauge isolate virulence may lead to more tailored treatment for canine hemorrhagic canine gastroenteritis and other *Clostridium*-mediated infections.

## Data Availability

The datasets supporting the conclusions of this article are available in the NCBI repository BioProject number PRJNA607435 http://www.ncbi.nlm.nih.gov/bioproject/607435.

## Abbreviations

Cpa: *Clostridium perfringens* alpha toxin
Cpb: *Clostridium perfringens* beta toxin
Etx: *Clostridium perfringens* epsilon toxin
Iap/Iab: *Clostridium perfringens* iota toxin
Cpe: *Clostridium perfringens* enterotoxin
NetB: *Clostridium perfringens* necrotic enteritis toxin B
NetE: *Clostridium perfringens* necrotic enteritis toxin E
NetF: *Clostridium perfringens* necrotic enteritis toxin F
TcsL: *Clostridium sordellii* lethal toxin
TcsH: *Clostridium sordellii* hemorrhagic toxic
pCS1: *Clostridium sordellii* plasmid 1
pCS2: *Clostridium sordellii* plasmid 2
Sdl: Sordellilysin
NanS: Neuraminidase S
Csp: Clostridium sordellii Phospholipase C
API: Analytic Profile Index
